# Replicate Phylogenies and Post-Glacial Range Expansion of the Pitcher-Plant Mosquito, *Wyeomyia smithii*, in North America

**DOI:** 10.1371/journal.pone.0072262

**Published:** 2013-09-06

**Authors:** Clayton Merz, Julian M. Catchen, Victor Hanson-Smith, Kevin J. Emerson, William E. Bradshaw, Christina M. Holzapfel

**Affiliations:** Institute of Ecology and Evolution, University of Oregon, Eugene, Oregon, United States of America; University of Arkansas, United States of America

## Abstract

Herein we tested the repeatability of phylogenetic inference based on high throughput sequencing by increased taxon sampling using our previously published techniques in the pitcher-plant mosquito, *Wyeomyia smithii* in North America. We sampled 25 natural populations drawn from different localities nearby 21 previous collection localities and used these new data to construct a second, independent phylogeny, expressly to test the reproducibility of phylogenetic patterns. Comparison of trees between the two data sets based on both maximum parsimony and maximum likelihood with Bayesian posterior probabilities showed close correspondence in the grouping of the most southern populations into clear clades. However, discrepancies emerged, particularly in the middle of *W. smithii*'s current range near the previous maximum extent of the Laurentide Ice Sheet, especially concerning the most recent common ancestor to mountain and northern populations. Combining all 46 populations from both studies into a single maximum parsimony tree and taking into account the post-glacial historical biogeography of associated flora provided an improved picture of *W. smithii*'s range expansion in North America. In a more general sense, we propose that extensive taxon sampling, especially in areas of known geological disruption is key to a comprehensive approach to phylogenetics that leads to biologically meaningful phylogenetic inference.

## Introduction

The term “resampling” in phylogenetics almost exclusively refers to bootstrapping or other methods of subsampling a single data set. Herein, we present a different kind of resampling: a two-stage phylogenetic analysis based on separate collections from near-by but geographically separate natural populations of the pitcher-plant mosquito, *Wyeomyia smithii*. We expected that using the same published methods, protocols and phylogenetic analyses would generate separate sets of phylogenetic trees whose major branches would align with each other. The previous study [1] was based on 21 populations; the present study was based on 25 populations. After comparing the two data sets, we merged the data into a combined set of 46 populations, which leads to new conclusions on the origin of northern and mountain populations of *W. smithii*. We then evaluated the validity of phylogenetic inference based on the 46-population maximum parsimony tree by comparing its topology with geological and historical biogeography of eastern North America.

*Wyeomyia smithii* is especially well suited for intraspecific phylogenetic comparisons because it consists of fully interfertile populations, which occur throughout an unusually broad range from the Gulf of Mexico to northern Canada (30–54°N). Mosquito populations are found only in the leaves of their carnivorous host plant, *Sarracenia purpurea*. *Sarracenia purpurea* is locally abundant but regionally scarce, thereby limiting gene flow among populations of this weak flying mosquito that is highly prone to desiccation [2]. *Wyeomyia smithii* overwinters in a state of larval diapause. All collections were made during the fall or early spring when 100% of each population and, hence, all of its genotypes are represented as overwintering larvae within the pitcher-plant leaves.

Earlier studies from our lab involving morphology, physiology, reproductive biology, allozymes and mtDNA clearly established sister clades along the Gulf of Mexico and the southern Carolina coastal plain, but left the relationships between these southern clades and more northern populations uncertain [1], [3]–[4]. Given the position of the Laurentide ice sheet at the last glacial maximum ca. 22,000–19,000 y ago [5]–[7], all present-day populations north of ca. 41°N latitude must have arisen within the last 19,000 y [7] from populations in the southern Appalachians or from populations existing along the glacial front. Our initial phylogeny [1] indicated that the post-glacial origin of northern populations resided in the ancestors of the southern Appalachian Mountain populations, followed by post-glacial migration to the Mid-Atlantic States and, subsequently, diverging to the northeast and the northwest. This second phylogeny was created for the dual purpose of testing the validity of the initial study [1] by sampling nearby but geographically separated populations and verifying distinct northeastern and northwestern clades by extending sampling to the northeast and northwest of previously sampled populations.

## Results

### Populations

The range of sampled North American populations extended from the Gulf Coast (30–31°N) to Newfoundland (50°N) and northwestward to Saskatchewan (54°N), from 30–4,000 ft elevation at 35°N in North Carolina (including Appalachian Mountain communities), and from 30–2,000 ft elevation at 40–41°N in New Jersey and the Pocono Mountains of Pennsylvania. Throughout the text, populations are referred to by their state or province of origin, followed by an identifying number when more than one population was sampled in a given state or province (Table S1). The latitude, longitude and altitude of each population as well as its identifying acronym are also provided in Table S1. At least 100 individuals from multiple pitcher-plant leaves were collected from each population and, in each case, wild-caught individuals were used for phylogenetic inference.

### Repeatability of Phylogenies

**Figure 1:**
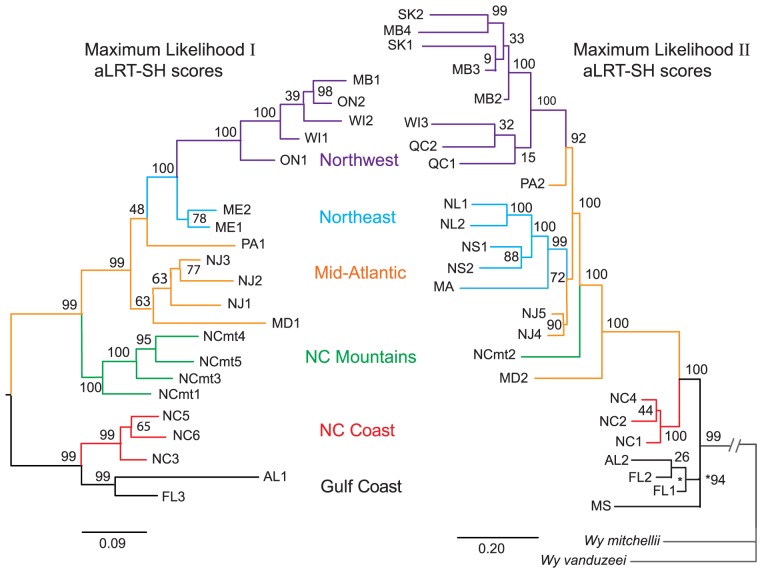
Replicability of maximum likelihood trees. **Left**, phylogenetic tree from [1] using 21 populations and 54 bp reads **Right**, phylogenetic tree from the current study using 25 populations and 80 bp reads. Branches leading to *W. mitchellii* and *W. vanduzeei* are abbreviated to clarify presentation. Color code indicates region of geographic origin. Numbers show nodal support as aLRT-SH scores. Two-letter abbreviations identify each state or province. Where two or more sites come from the same state or province, they are identified by number. Latitudes, longitudes, altitudes, and acronyms connecting the specific localities with earlier studies from this lab are provided in Table S1. The scale bars indicate substitutions per site.

**Figure 2:**
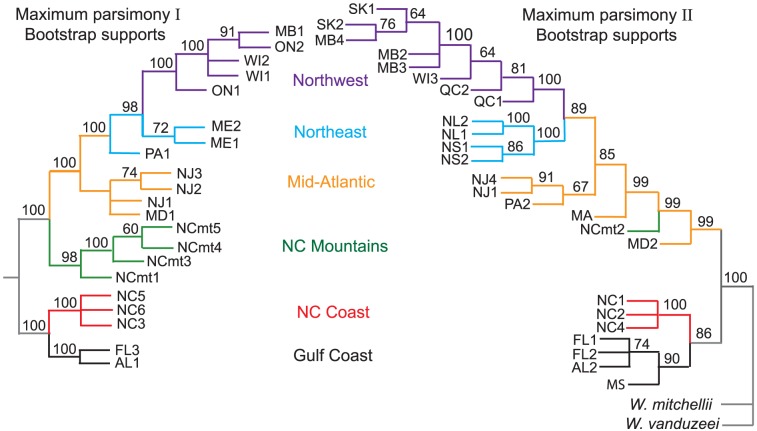
Replicability of maximum parsimony trees. **Left**, phylogenetic tree from [1] using 21 populations and 54 bp reads. **Right**, phylogenetic tree from the current study using 25 populations and 80 bp reads. Colors and abbreviations as in Figure 1. Nodal values show bootstrap support >50%.

**Figure 3:**
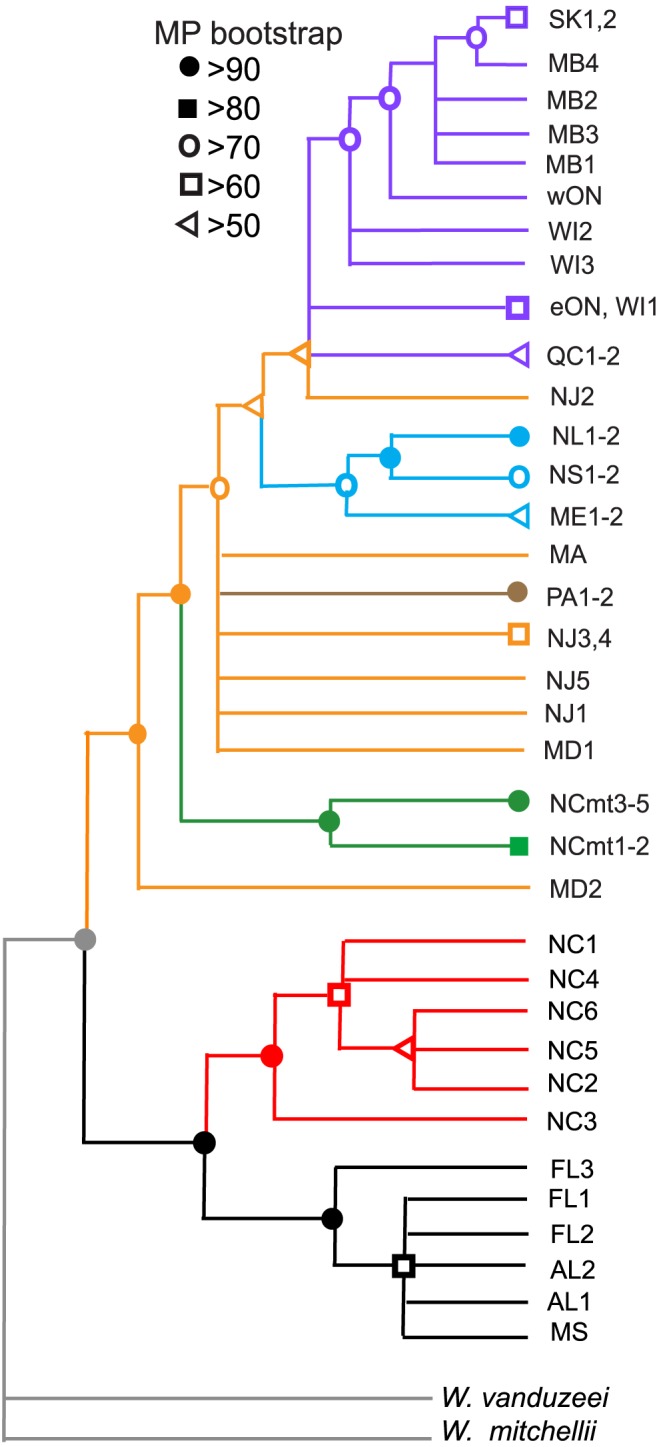
Maximum parsimony with bootstrap support for the combined 46 populations. The tree is based on 54>50% support. Color codes are the same as in Figures 1–2. There are two major monophyletic groupings: one consisting of populations along the Gulf and North Carolina Coasts, the other consisting of all more northern and mountain populations. The mainland Maryland population (MD2) shares a most recent common ancestor with all more northern and mountain populations. Phylogenetic results generally follow latitude except for the mountain populations (NCmt, PA). There are six well supported clades (bootstrap>70) that include two or more populations: Gulf Coast (MS, AL, FL: black), North Carolina Coast (NC: red), North Carolina mountains (NCmt: green), Pocono Mountains (PA: brown), northeast (ME, NS, NL: blue), and northwest (WI2–3, wON, MB, SK: purple). The North Carolina mountain populations (NCmt) split into two clades: one in the Savannah River drainage leading to the Atlantic Ocean (NCmt1–2), the other in the Tennessee River drainage (NCmt3–5) leading to the Gulf of Mexico.

Figures 1–2 compare the initial (left) [1] and second (right) phylogenies. Trees from both studies were rooted using *Wyeomyia mitchellii* and *W. vanduzeei* as outgroups. The initial trees (left) used COI data to form a tree with no rooting constraints, then used the results of that tree to indicate which populations should be the root of the RAD sequence tree [1]. The second set of trees (Figs. 1–2) and the combined tree (Fig. 3) used the RAD data alone to produce the trees without constraining a root beforehand. Both the initial and second set of trees show that the southern populations (Gulf Coast & North Carolina Coast) form two well-supported clades that share a most-recent common ancestor (MRCA) with all more northern and mountain populations of *W. smithii*. Figure 1 reports approximate likelihood ratio tests with non-parametric Shimodaira-Hasegawa support measures (aLRT-SH) [8]–[9] scaled from 0 to 100 with Bayesian posterior probabilities; Figure 2 reports maximum parsimony with bootstrap support as these values were the units used in the previous phylogenies [1]. There is close general correspondence between groupings of the most southern populations along the Gulf and southern North Carolina coasts. The sharpest differences occur in the positions of the Maryland (MD), North Carolina mountain (NCmt), and Pocono mountain (PA) populations, which are placed at different positions among the four trees.

### Combined Phylogeny

The combined phylogeny (Fig. 3) clearly clusters Gulf Coast, southern North Carolina Coast, North Carolina mountain, Pocono mountain, Northeast and Northwest populations into distinct clades. All but the Gulf and southern North Carolina Coast clades share a most-recent common ancestor within the Mid-Atlantic populations. Specifically, the mainland Maryland population (MD2) shares a most recent common ancestor with all more northern populations and with populations in the North Carolina mountains as well. The Maine, Nova Scotia and Newfoundland populations form a distinct Northeast clade; the sequence of populations from southern and western Wisconsin (WI2,3), to western Ontario (wON), to northern Manitoba (MB1–4) and to eastern Saskatchewan forms a clear Northwest clade. The North Carolina mountain populations split into two clades. One clade resides in the Savannah River drainage leading to the Atlantic Ocean (NCmt1–2); the other clade resides in the Tennessee River drainage leading to the Gulf of Mexico (NCmt3–5).

## Discussion

Our expectation when constructing a second set of phylogenies was that at least the major branches of the trees would align with each other, whether represented by single or multiple populations. This expectation proved to be the case in the southern portion of *W. smithii*'s range where all trees illustrate well supported phylogenies that resolve the same distinct clades (Figs. 1–2). However, when the initial (left) and second (right) independent phylogenies are compared, a number of uncertain relationships emerge, primarily relating to populations at intermediate latitudes. From the earlier trees (Figs. 1–2, left): The mountain populations from North Carolina shared their most recent common ancestor (MRCA) with all the more northern populations; the Maryland population (MD1) shared a MRCA with the New Jersey (NJ) populations; the Pocono Mountain population (PA1) shared a MRCA with all more northern populations; and, the Maine populations (ME) shared a MRCA with all populations to the northwest. The relationship of the Maine populations to the northwest populations makes it unclear whether there is a distinct northeast clade or whether populations from even more northeastern localities would merge into a single northern clade. By contrast, from the recent trees (Figs. 1–2, right): the Maryland population (MD2) was basal to all more northern populations and to the North Carolina mountain population (NCmt2); the position of the Pocono Mountain populations (PA1 vs. PA2) differed between the trees; and, there emerged a distinct northeast clade sharing its MRCA within the Mid-Atlantic populations. Therefore, had we depended only on our first phylogeny as the basis for all our future evolutionary studies, we would have had a well-supported but incomplete vision of *W. smithii'*s evolutionary history.

We next combined both data sets to create a combined three. This tree (Fig. 3) was based on the first 54 bp after the *Sbf1* cut site for all populations and permitted the two independent data sets to confront each other as a unit. The combined tree included 46 populations, approximately doubling the number of populations in any of the initial 54 bp or 80 bp trees taken singly. The combined tree (Fig. 3) coalesced the North Carolina mountain populations into a single clade and the Pocono Mountains populations (PA) into a single clade while leaving separate the two Maryland (MD) populations. The Eastern Shore Maryland population (MD1) is historically, geologically and geographically separated by the Chesapeake Bay from the important mainland Maryland population (MD2). The Chesapeake Bay itself was formed about 10Kya when rising sea levels flooded the Susquehanna River valley [10]–[12], resulting in the separation of the sandy peninsular MD1 population from the mainland MD2 population. The mainland (MD2) population is pivotal to the post-glacial range expansion of *W. smithii* and shares a MRCA not only with all more northern populations, but with the North Carolina mountain populations as well. The importance of the MD2 population is apparent both in the 80 bp trees and in the combined tree after the MD2 reads had been trimmed to 54 bp; in each case, MD2 retains its position as having a MRCA with all more northern and mountain populations.

**Figure 4:**
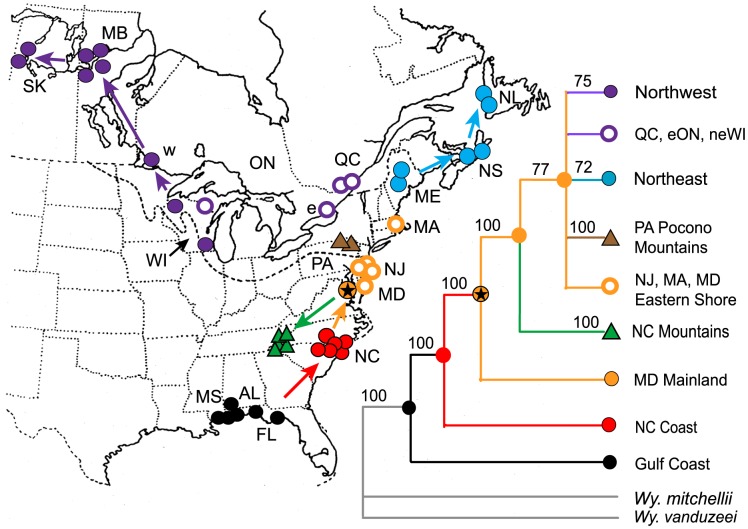
Phylogeography of *Wyeomyia smithii* based on the combined 46-population tree (**Fig. 3**), on historical biogeography, and on the timing of glacial events. Arrows indicate directions of range expansion based on Figure 3. Maximum extent of the Laurentide Ice Sheet at the last glacial maximum is plotted as a dotted line [6]. Two-letter abbreviations identify each state or province. Color codes are the same as in Figures 1–3. Map symbols represented by a triangle show mountain populations in the southern Appalachians of North Carolina (green) or in the Pocono Mountains of Pennsylvania (brown). The filled orange symbol with the star indicates the mainland Maryland population (MD2). Numbers associated with each node or branch tip represent maximum parsimony bootstrap support for that clade. There are no support numbers for the QC, eON and neWI or the NJ, MA, and Eastern Shore MD populations (open circles) because they do not constitute a monophyletic grouping. e, eastern Ontario (ON1); w, western Ontario (ON2).

Based on current sampling, these results lead us to conclude that post-glacial range expansion of *W. smithii* proceeded from populations near the glacial front (Fig. 4), not from the southern Appalachian Mountains in North Carolina as we had concluded earlier [1] and that the origin of all northern and Appalachian mountain populations arose from common ancestors of what is now the mainland Maryland population (MD2). In many ways, phylogenies are informed hypotheses and, regardless of the detailed methodologies of phylogenetic inference, biology still matters. Consequently, we ask the important question: Does the known post-glacial geology and historical biogeography of eastern North America support the phylogenetic relationships in Figure 3?

### Geological and Historical Biogeography

The Laurentide Ice Sheet and its recession have had dramatic consequences on vegetation formation and biomes during the last 20,000 years [13]–[17]. There is a wealth of temporal information on the distribution of plants during this period and we can ask how well the phylogenetic inference of northern *W. smithii* populations (Fig. 3) aligns with the historical biogeography of these plants. *Wyeomyia smithii* is a single species of fully interfertile populations throughout its range and lives only in the water-filled leaves of a single species of carnivorous plant, *Sarracenia purpurea*. *Sarracenia purpurea* has historically been found in *sphagnum* peatlands and, in the northern parts of its range, is associated with tamarack (*Larix larcina*) and black spruce (*Picea mariana*) [18]. We have used the co-occurrence of *Sphagnum*-dominated peatlands and these two tree species as reliable indicators for the presence of pitcher plants while searching for new populations of *W. smithii* over the last 40 years. During the last glacial maximum (ca 20–22 Kya), *Sphagnum*-dominated peatlands east of the Appalachian Mountains ranged from northern North Carolina lowlands to southern Maryland, followed the glacial retreat first northeastward 26-18 Kya, then westward south of the current Great Lakes 12–14 Kya, and finally northwestward 8–10 Kya [14]–[15] approximating the draining of Lake Agassiz [19]. The pattern of post-glacial colonization of tamarack and spruce closely followed that of these *Sphagnum* peatlands [14], [16]. Therefore, the historical biogeography of *Sphagnum* peatlands and their associated flora provide independent evidence (1) for persistence of pitcher plants and *W. smithii* near the glacial front and (2) for the formation of separate northeastern and northwestern clades as shown in the combined phylogeny (Fig. 3).

Figure 4 illustrates our interpretation of post-glacial dispersal of *W. smithii* based on data from the combined tree (Fig. 3) and on the historical biogeography of the flora associated with its host plant, *Sarracenia purpurea* during the last glacial maximum and during the sequential retreat of the Laurentide Ice Sheet. *Wyeomyia smithii* persisted through the last glacial maximum near the glacial front and the mid-Atlantic populations represent the longest established populations north of the Carolinas. We propose that the lack of well supported nodes among the Mid-Atlantic populations is due to gene flow in a group of geographically proximal populations compressed near an advancing and receding glacial front for thousands of years.

With recession of the Laurentide Ice Sheet, populations near the glacial front served as a source for populations in the southern Appalachians and for populations dispersing into the Mid-Atlantic region. Mid-Atlantic populations then spread upward into the Pocono Mountains of Pennsylvania, northeastward along the coast and proximal inland localities to Newfoundland, and later westwards. The timing of glacial melt and historical biogeography indicate that the likely route of westward migration occurred to the south of the Great Lakes, thence northwestwards. This interpretation is supports the southern and western Wisconsin to Saskatchewan phylogenetic relationships of the Northwest clade (Fig. 3). At a later time, *Sphagnum* peatlands were established north of the Great Lakes [14]–[15] and this more recent establishment provides a rationale for the lack of greater node support for direction of evolution among the Québec, eastern Ontario, and northeastern Wisconsin populations (Fig. 3). Hence, geological and historical biogeography parallels the phylogeny inferred in Figure 3. In combination, the inferred phylogeny supported by geological and historical biogeography lead us to the phylogeography illustrated in Figure 4.

### Improving the Selection of Populations

Both the initial (Figs. 1–2, left) and second (Figs. 1–2, right) trees individually had well supported branches. Yet, when compared, there was disparity between them among populations, particularly in the Mid-Atlantic region. Merging the two data sets clustered several individual branches into distinct clades and identified the Mid-Atlantic region as the source of the entire post-glacial dispersal of populations into northern regions. Hence, the combined tree (Fig. 3) enabled us to clarify issues raised by comparisons of the two trees (Figs. 1–2). We have put considerable thought into how we could have increased the efficiency of this process. The time and cost of collecting independent populations on different trips far exceeded the time and cost of sequencing and identifying thousands of informative SNPs. Our work with *W. smithii* tells us that we would have achieved improved phylogenetic inference more efficiently by collecting populations more intensively over the species' range, focusing most deliberately on the populations likely to have encountered historical or geological barriers to migration. In our case, these barriers would certainly include the southern boundary of the Laurentide Ice Sheet (Fig. 4, dashed line) and, secondarily, Lake Agassiz and the Great Lakes regions. The goal should have been to collect from as many discrete natural populations as possible, including nearby replicate populations, even if seemingly excessive at the outset. Since samples can be stored at −80°C indefinitely, a single collecting trip would have provided a library of populations for both current and future projects. We would then have made our first trees (Figs. 1–2) closer to our combined tree (Fig. 3) based on our knowledge of the organism and its known geographical history to answer our initial questions. We could then have drawn from our frozen library additional populations from regions of unexpected phylogenetic uncertainty and from regions appropriate for answering new and different questions.

## Methods

### Ethics Statement

We are grateful to the Gros Morne National Park, The Nature Conservancy, the North Temperate Lakes LTER, the Cheraw State Park, the Weymouth Woods-Sandhills Nature Preserve, the Tobyhanna State Park, the Grand Bay National Estuarine Research Reserve, and the Highlands Biological Station for assistance in making collections on their lands. Both the Centers for Disease Control and the Department of Agriculture were most helpful in arranging import permits for *W. smithii* from Canada.

### RAD Library Creation and Sequencing

Wild-collected populations were frozen at −80°. Six individuals were removed haphazardly from the frozen animals and pooled for DNA extraction into a single sample for each population.

Both the initial and replicate phylogenies used single-nucleotide polymorphisms (SNPs) located within thousands of loci generated from restriction-site associated DNA (RAD) sequences. To maximize an apples-to-apples comparison, both phylogenies used the same restriction enzyme, pipeline, and, methodologies for phylogenetic inference [1], except the first was based on 54 bp reads and the second on 80 bp reads. The two datasets were first treated separately and then merged. When the two datasets were merged, the 80 bp data were trimmed to the 54 bp immediately following the *Sbf1* restriction site to avoid over-weighting the second data set in the combined analysis.

Each population's relative DNA concentration was measured fluorimetrically with an intercalating dye prior to sequencing. The DNA concentration for each population's RAD library was used to create a combined solution for each lane of sequencing. Each lane contained an equal amount of DNA from each population. Fluorimetry was again used to measure the DNA concentration for each lane, which was then diluted to obtain equal DNA concentrations for each lane. Three libraries were single-end sequenced in three lanes of an Illumina GAIIx (Table S2), resulting in a total of more than 94 million RAD sequences of which more than 41 million sequences passed several levels of quality filtering. The 25 populations of *W. smithii* and the two outgroups (*W. mitchellii* and *W. vanduzeei*) were represented by an average of 1,485,071±157,646 SE sequences. Within each population, we identified an average of 50,853±6,854 SE stacks spread across 43,009±6,106 SE loci, resulting in an average of 1.18 stacks per RAD tag locus (Table S2).

We again used the *Stacks* pipeline [20] to identify sequence sites that were fixed within individual populations, but variable among populations. The programs *process_radtags*, *ustacks*, and *hstacks* are all embedded within *Stacks*. We executed the *process_radtags* program to remove potentially erroneous raw reads from the datasets and to de-multiplex the reads into population samples. The *process_radtags* program uses a sliding window analysis that allows for isolated errors in the raw Illumina data but discards reads in which a sustained drop in quality is detected along the read length. Each population sample was then processed using the *ustacks* program. The *ustacks* program groups all exactly matching sequences into stacks and then forms loci from sets of stacks. We set the parameters for *ustacks* such that for each stack in the locus there was another stack in the locus that is at most one nucleotide divergent (-M 1). We also specified that a minimum of three raw reads were required to form a stack (-m 3). We used the *fixed* maximum likelihood model (-T fixed) to identify nucleotides within each locus that were fixed homozygous [1]; nucleotide positions that could not be identified as fixed were masked out and replaced with “N” characters. The *fixed* model requires an estimated error rate (-e). We estimated the sequencing error rate by counting the number of barcodes that were known to be erroneous. The new populations were sequenced in three lanes, resulting in the following three error rates (the fraction of incorrectly called nucleotides from the total number of nucleotides within sequenced inline barcodes), one per sequencing lane: **0.0623:** *Wyeomyia vanduzeei* and *W. smithii* populations AL2, NCmt2, MD2, NS1, NL2, QC1, WI3, SK1; **0.0712:** *W. mitchellii* and *W. smithii* populations MS, MA, NS2, NL1, QC1, MB4, SK2; **0.0865:** *W. smithii* populations FL1, FL2, NC1, NC2, NC4; NJ4, NJ5, PA2, MB2, MB3. Finally, all samples were fed into the *hstacks* program, which matched loci across populations, allowing for at most one fixed difference between the populations (-n 1). We then used the *extract_interpop_chars.pl* program in the *Stacks* pipeline to extract nucleotides from the final matched loci that, while fixed within each population, varied among at least two populations. Once a fixed-within and variable-among SNP is identified, it is extracted from the RAD locus and inserted into a FASTA file, without surrounding sequences. The final result is a FASTA file of concatenated fixed-within and variable-among population SNPs. The numbers of fixed within- and variable among-population SNPs were 3,741 for the 54 bp ML tree and 16,858 for the 80 bp ML tree. The number of parsimony informative sites were 1,472 for the 54 bp tree, 3,172 for the 80 bp MP tree and 3,286 for the combined MP tree after the 80 bp reads were trimmed to 54 bp.

### Phylogenetic Inference

In parallel with our previous RAD-seq study [1], we inferred phylogenies using maximum parsimony (MP) with bootstrap support and maximum likelihood (ML) with approximate likelihood ratio (aLRT-SH). MP trees were found using PAUP* [21] with a standard heuristic search. Support for branches on the MP tree was estimated using a bootstrap with 200 samples. ML trees were developed in three steps: model selection, model optimization, and branch support estimation. We first found the best-fitting nucleotide model using a hierarchical likelihood ratio test (hLRT) and the Akaike information criterion (AIC) as implemented in jModelTest [22] and ModelTest [23]. For the initial and new data set, the hLRT and AIC agreed that the best-fitting model was the transversion model (TVM) with variable base frequencies, variable transversion rates, and transition rates equal. We next found ML trees by optimizing the parameter values and branch lengths of the best-fitting models using PhyML version 3.0 [8]. Finally, we estimated support values on the ML trees using a non-parametric Shimodaira–Hasegawa-like approximate likelihood ratio test (SH-like aLRT) [9] scaled from 0 to 100. SH-like aLRTs were used in order to make parallel comparisons with the previously-published ML tree [1].

### Combined Phylogeny

Genomic loci that are not resolved within local populations can lead to spurious inferences about relationships among global populations. We minimized this problem by removing loci from our analysis using a custom maximum likelihood model that kept only those sites that were inferred to be homozygous within each sample [24]. This model is not reliant on diploid individuals, thereby assuring that each site used in our analysis was indeed fixed within each population.

Incomplete lineage sorting can cause some loci to have evolved according to speciation patterns that are discordant with other loci; incomplete lineage sorting can result in gene trees that are not in accord with the implied species trees [25]–[26]. To mitigate discordance, both studies sampled sites broadly from across each genome. Specifically, the *Sbf1* restriction enzyme binding sites (i.e. RAD sites) are scattered across genomes at random locations [27]. Random scattering means that *Sbf1* sites are not biased *a priori* towards particular linkage groups, gene clusters, or genomic regions from correlated evolutionary histories. To the extent that a dominant pattern of evolutionary divergence underlies the populations in our study, our use of thousands of *Sbf1* sites should recover the majority of this evolutionary history. Second, to mitigate discordance, we used multiple individuals per population as suggested by Degnan and Rosenberg [25]. Third, we based phylogenetic inference of the combined tree on maximum parsimony (MP) that makes no explicit assumptions other than that the tree requiring the fewest substitutions is the best tree. For *W. smithii*, we selected only those sites that were monomorphic within populations and polymorphic among populations. Hence, our concatenated data set most closely fits the assumptions of MP in which a nucleotide site is parsimony-informative only when there are at least two different kinds of nucleotides at a site, and each of these nucleotides is represented by at least two of the sequences being considered [28]–[30]. Finally, with concatenated data, MP more frequently estimates the true species tree than maximum likelihood, especially when a large number of genes is evaluated, even though MP may be biased in the presence of long-branch attraction [31].

### Data Accessibility

All raw sequence reads are available at the NCBI Short Read Archive, the first data set by accession number SRA012678.1 and the second by SRA01472.1.

## Supporting Information

Table S1 **Origin of populations.**
(XLSX)

Table S2 **Stacks statistics for the 80 bp tree.**
(XLS)
